# Minimal-invasive anterior approach to the hip provides a better surgery-related and early postoperative functional outcome than conventional lateral approach after hip hemiarthroplasty following femoral neck fractures

**DOI:** 10.1007/s00402-022-04602-2

**Published:** 2022-09-02

**Authors:** M. Orth, D. Osche, P. Mörsdorf, J. H. Holstein, M. F. Rollmann, T. Fritz, T. Pohlemann, A. Pizanis

**Affiliations:** 1 Department of Trauma, Hand and Reconstructive Surgery, Saarland Univesity, Kirrberger Strasse 1, D-66421 Homburg, Saarland Germany; 2 Ethianum Clinic, Heidelberg, Germany; 3 BG Klinik Tuebingen on behalf of the Eberhard-Karls-University Tuebingen, Tuebingen, Germany

**Keywords:** Hip, Femoral neck fracture, Hemiarthroplasty, AMIS, Lateral approach

## Abstract

**Introduction:**

Femoral neck fractures (FNF) are one of the most frequent fractures among elderly patients and commonly require surgical treatment. Bipolar hip hemiarthroplasty (BHHA) is mostly performed in these cases.

**Material and methods:**

In the present retrospective study geriatric patients with FNF (*n* = 100) treated either by anterior minimal-invasive surgery (AMIS; *n* = 50) or lateral conventional surgery (LCS; *n* = 50) were characterized (age at the time of surgery, sex, health status/ASA score, walking distance and need for walking aids before the injury) and intraoperative parameters (duration of surgery, blood loss, complications), as well as postoperative functional performance early (duration of in-patient stay, radiological leg length discrepancy, ability to full weight-bearing, mobilization with walking aids) and 12 months (radiological signs of sintering, clinical parameters, complication rate) after surgery were analyzed.

**Results:**

Patients in the AMIS group demonstrated a reduced blood loss intraoperatively, while the duration of surgery and complication rates did not differ between the two groups. Further, more patients in the AMIS group achieved full weight-bearing of the injured leg and were able to walk with a rollator or less support during their in-patient stay. Of interest, patients in the AMIS group achieved this level of mobility earlier than those of the LCS group, although their walking distance before the acute injury was reduced. Moreover, patients of the AMIS group showed equal leg lengths postoperatively more often than patients of the LCS group. No significant differences in functional and surgery-related performance could be observed between AMIS and LCS group at 12 months postoperatively.

**Conclusions:**

In conclusion, geriatric patients treated by AMIS experience less surgery-related strain and recover faster in the early postoperative phase compared to LCS after displaced FNF. Hence, AMIS should be recommended for BHHA in these vulnerable patients.

## Introduction

Femoral neck fractures (FNF) are one of the most frequent fractures among elderly patients and commonly require surgery [1]. However, the surgical treatment causes a perioperative stress and may thereby have a detrimental effect on patients especially in cases with a reduced health status before the acute injury [2]. Therefore, surgery-related stress on the trauma patient should be reduced to a minimum [3]. In cases of non- or only slightly displaced FNF several femoral head-preserving implant systems such as e.g. cannulated screws and others are available and can be applied by minimal-invasive, quick procedures [4]. In contrast, displaced FNF commonly require arthroplasty of the hip joint. Arthroplasty is known to be considerably more invasive and more strenuous for the patient [5]. To reduce the strain on the patient, bipolar hip hemiarthroplasty (BHHA) instead of total hip arthroplasty can be performed. BHHA replaces the femoral head and neck with an artificial component that can be inserted into the femoral canal (with or without cement). The bipolar concept of this type of hemiarthroplasty is based on a double sphere system, of which the outer component articulates with the patient’s native acetabulum. The main mobility of this implant occurs via a multidimensional motion between the outer component, the innermost femoral head of the implant and the polyethylene inlay between these components. This procedure is conventionally performed via a lateral approach. However, in elective total hip arthroplasty (THA) the anterior minimal-invasive surgical approach has recently shown to be more beneficial for the patients than the lateral approach perioperatively and in the early postoperative phase by reducing blood loss and the duration of hospital stay [6]. We herein hypothesize that anterior minimal-invasive surgery (AMIS) is more beneficial in the perioperative and early postoperative phase after BHHA following displaced FNF than the lateral conventional surgery (LCS). For this purpose, we analyzed surgery-related and early postoperative functional outcome parameters retrospectively in patients receiving BHHA via these two different surgical approaches.

## Materials and methods

### Study design

This study was carried out as a comparative retrospective study in a single major trauma center. The patients of the study cohort suffered from a non-pathological, displaced FNF (type III or IV according to the Garden classification) and were surgically treated between February 2016 and December 2021. Depending on the surgical approach, patients were divided into two groups: the AMIS group, in which patients received BHHA by an anterior, minimal-invasive surgical approach, and the LCS group, in which patients received BHHA by a lateral surgical approach.

### Patient characterization

To characterize and to determine the health status of the selected patients before the FNF the following characteristics of the patients were obtained:

- Age at the time of surgery [year]
- Sex [female; male]
- Preoperative health status according to the American Society of Anaesthesiologists (ASA) physical function class [7] dividing the health status into five categories [I: healthy; II: light general illness; III: severe general illness; IV: permanent threat of life; V: moribund patient]
- Walking distance before the injury [I: unlimited; II: limited to 500 m; III: limited to 50 m; IV: indoor only; V: immobile]
- Need for walking aids before the injury [I: none; II: forearm crutches; III: rollator / walking frame; IV: wheelchair; V: bedridden]

### Surgical procedures for hip hemiarthroplasty

In the present study, bipolar hemiarthroplasty of the hip after FNF was performed either via a lateral surgical approach or an anterior minimal-invasive approach. The choice of the approach was random and the interventions were performed by 15 consultants of the department in total with a random distribution of the performing surgeons between the two groups.

**Fig. 1:**
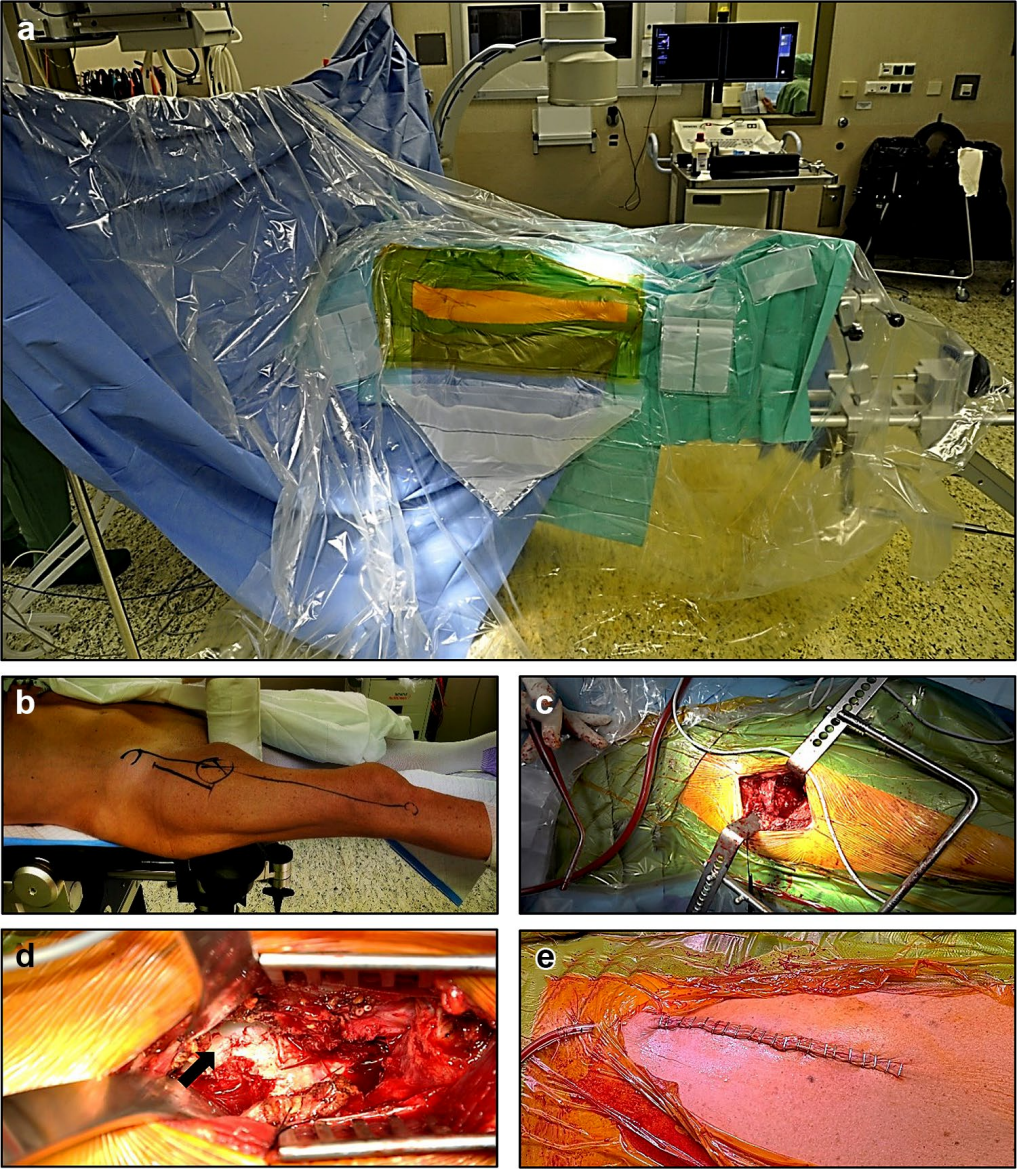
Minimal-invasive surgical technique for hip hemiarthroplasty following femoral neck fractures. **a:** Supine position of the patient on an extension table mounting the injured leg on a traction/reduction system. **b:** Marking the longitudinal incision of approximately 8 cm at the proximal lateral thigh beginning 3–4 cm lateral and 3–4 cm distal to the anterior superior iliac spine and pointing towards the patient’s fibular head in line with the course of the tensor fascia lata muscle (TFL). **c:** Insertion of a retractor to widen the interval between non-injured rectus femoris and iliopsoas muscles medially and gluteus medius muscle laterally to expose the anterior joint capsule. **d:** Capsulotomy performed to identify the femoral head (arrow) and neck area. **e:** Wound closure completes the procedure after hemiarthroplasty

The anterior minimal-invasive surgical approach is performed in the supine position of the patient on an extension table with the injured leg being mounted on a traction and reduction system (Fig. 1). After incision at the proximal lateral thigh the fascia overlying the tensor fascia lata muscle (TFL) is incised longitudinally and further blunt preparation is performed without cutting muscle fibres. Branches of the lateral femoral circumflex artery and vein are isolated and ligated and after exposure of the anterior joint capsule a capsulotomy in a flap shape is then performed to identify the femoral head and neck including the fracture site (Fig. 1).

The lateral surgical approach to the hip is performed in the lateral position in the present study. After incision over the greater trochanter the fascia is incised longitudinally over the prominence of the greater trochanter. The underlying muscles are dissected in line with its fibers and after capsulotomy the fracture and the femoral neck are exposed.

After exposure and rasping implantation of the bipolar hemiarthroplasty can be performed. For AMIS, specially designed, commercially available instruments are needed to access the medullary canal, whereas LCS requires new positioning of the injured leg by crossing it over the other patient’s leg. In the present study, all BHHA were implanted with cement (implants used for AMIS: Medacta International, Castel San Pietro, Switzerland; implants used for LCS: MS-30 by Zimmer Biomet, Warsaw, United States). Eventually, wound closure completes the procedure (Fig. 1e).

### Intraoperative and early postoperative functional performance during and after surgery

The following parameters were used to analyze surgery-related parameters during and after treatment of either AMIS or LCS:

- Duration of surgery [minutes]
- Blood loss [ml] according to the surgeon’s documentation based on the suction collection container at the end of surgery
- Difference between the level of hemoglobin (Hb) at admission minus level of Hb in the first blood sample after surgery [g/dl]
- Difference between the level of creatine kinase (CK) in the first blood sample after surgery [U/l] minus level of CK at admission
- Difference between the level of C-reactive protein (CRP) in the first blood sample after surgery [mg/l] minus level of CRP at admission
- Type of postoperative monitoring obligation [Intensive Care Unit (ICU); Intermediate Care Unit (IMC); Normal ward]
- Categorized intraoperative or early postoperative complications [None; wound infection; luxation of the operated hip joint; iatrogenic fracture of the femur; sintering of the implant in postoperative X-ray controls; others not related to the surgery]

The postoperative functional outcome after treatment with BHHA by either AMIS or LCS was analyzed by using the following parameters. To focus on the early postoperative outcome, we used data from the in-patient stay for this analysis only:

- Duration of in-patient stay [day]
- Radiological leg length discrepancy [mm]. Differences of leg length of − 5 mm to + 5 mm compared to the contralateral side were considered equal. Differences of either more or less than 5 mm compared to the contralateral side were considered unequal.
- Ability to full weight-bearing of the injured extremity during in-patient stay [yes/no]
- Mobilization to standing position during in-patient stay [postoperative day]
- Mobilization with rollator or less support during in-patient stay [possible/not possible]. Of those, who were able to walk with a rollator or less support, the postoperative day was analyzed [postoperative day]
- Need for walking aids at discharge [I: none; II: forearm crutches; III: rollator / walking frame; IV: wheelchair; V: bedridden]

The functional and surgery-related outcome 12 months after treatment with BHHA by either AMIS or LCS was analyzed by using the following parameters:

- Radiological sintering [mm]: To evaluate potential sintering of the implant after mobilization, different positions of the prosthetic stem in relation to the femur of more than 3 mm compared to X-rays directly after surgery was considered to be positive. Differences of less than 3 mm compared to the contralateral side were considered to be equal.
- Ability to full weight-bearing of the injured extremity [yes / no]
- Mobilization to standing position possible [number of patients]
- Walking on stairs possible [number of patients]
- Need for walking aids [None; improved compared to early postoperative results during in-patient stay]
- Categorized postoperative complications within the first 12 months after surgery [None; surgery-related such as e.g. infection or luxation; others not related to the surgery]

### Statistics

Power analysis was performed a priori to estimate the sample size using an effect size of 0.75 (G*Power, University Kiel, Germany). Further statistical analysis was performed using the SPSS software (SPSS Statistics 28.0; IBM Corporation, Armonk, USA). General descriptive analysis was carried out using mean ± standard error of the mean (SEM) for continuous variables. Comparison of continuous variables was first tested for normal distribution and equal variance and comparison between the groups was performed by Mann–Whitney *U* test. The chi-square test (SPSS Statistics 28.0) or Fisher’s exact test (SPSS Statistics 28.0) were used to compare the groups followed by a post hoc test including the correction of the α-error according to Bonferroni probabilities (Microsoft Excel 2019, Microsoft, Redmond, USA) to compensate for multiple comparisons in cases of statistical significance to determine the specific statistically significant categorical variable. A *p* value < 0.05 was considered to indicate significant differences.

## Results

### Patient characterization

In total 100 patients were included in this study (Table 1). Both groups consisted of 50 patients each (AMIS: 38 female and 12 male; LCS: 32 female and 18 male). The mean age at the time of surgery was 82.5 years (AMIS) and 79.9 years (LCS), respectively. The ASA score as a parameter for the physiological status of each patient showed that the majority of the included patients (*n* = 96/100) suffered from mild (ASA II) to severe (ASA III) systemic diseases (AMIS: ASA II: *n* = 11, ASA III: *n* = 37; LCS: ASA II: *n* = 20, ASA III: *n* = 28). None of these preoperative parameters (age, sex, ASA score) were statistically significant (Table 1). In contrast, the walking distance before the acute injury was unlimited in only 5/50 patients in the AMIS group, whereas 21 patients belonged to this category in the LCS group. Accordingly, patients of the LCS group showed a significantly better walking distance preoperatively. Although more patients of the LCS group did not need walking aids before the injury (*n* = 28) and half of all patients in the AMIS group (*n* = 25) were bound to a rollator or were even more compromised before the injury, the need of walking aids did not show significant differences between the two groups (Table 1).

**Table 1:** Characterization and preoperative health status of patients suffering from femoral neck fracture (FNF) and treated by either anterior minimal-invasive surgery (AMIS) or lateral conventional surgery (LCS) for hip hemiarthroplasty

	AMIS (*n* = 50)	LCS (*n* = 50)	*p* value
Age at time of surgery [years]	82.5 ± 0.96 (62–103)	79.9 ± 1.15 (60–98)	0.28^a^
Sex			0.19^b^
Female	38	32	
Male	12	18	
ASA score			0.09^b^
I:	0	1	
II:	11	20	
III:	37	28	
IV:	1	1	
V:	0	0	
Walking distance before injury [m]			0.01^b^
Unlimited	5*	21*	
Limited to 500 m	11	9	
Limited to 50 m	15	11	
Indoor only	10	5	
Immobile	8	4	
Need for walking aids before injury			0.40^b^
None	19	28	
Forearm crutches	5	6	
Rollator/walking frame	19	12	
Wheelchair	2	2	
Bedridden	4	2	

Mean ± SEM (range); *p < 0.05

*ASA* American Society of Anaesthesiologists

^a^Mann–Whitney *U* test

^b^Chi-square test/Fisher exact test

### Surgery-related analysis

The analysis of intraoperative and surgery-related parameters exhibited significantly reduced blood loss in patients undergoing AMIS compared to LCS (Table 2). In addition, the postoperative decrease of Hb and increase of CK was significantly different between patients of the AMIS group and patients of the LCS group (Table 2). In contrast, other direct surgery-related parameters such as the duration of surgery, which was approximately 1.5 h in both groups, and expression of CRP did not differ (Table 2). The necessity for postoperative monitoring obligation revealed that postoperative admission of most patients was on an ICU ward in both groups (AMIS: 34; LCS: 37; *p* > 0.05). Further analysis of the intraoperative or early postoperative occurrence of complications revealed that the majority of patients did not suffer from any surgery-related complications (Table 2).

**Table 2:** Analysis of surgery-related parameters of patients suffering from femoral neck fracture (FNF) and treated by either anterior minimal-invasive surgery (AMIS) or lateral conventional surgery (LCS) for hip hemiarthroplasty

	AMIS (*n* = 50)	LCS (*n* = 50)	*p* value
Duration of surgery [min]	86.9 ± 2.7	90.7 ± 3.6	0.53^a^
Blood loss	72.5 ± 11.2*	155.4 ± 17.3	< 0.001^a^
Difference Hb	1.8 ± 0.2*	2.4 ± 0.2	0.04^a^
Difference CK	156.8 ± 32.8*	304.25 ± 57.4	0.004^a^
Difference CRP	45.4 ± 6.7	55.6 ± 9.1	0.88^a^
Postoperative ward			0.66^b^
ICU	34	37	
IMC	1	0	
Normal ward	15	13	
Complications			0.32^b^
None	34	31	
Infection	1	0	
Luxation	0	1	
Iatrogenic fracture	0	3	
Sintering	0	0	
Others not related to the surgery	14	15	

Mean ± SEM; *p < 0.05

*Hb* hemoglobin, *CK* creatine kinase, *CRP* C-reactive protein, *ICU* Intensive Care Unit, *IMC* Intermediate Care Unit

^a^Mann–Whitney *U* test

^b^Chi-square test/Fisher exact test

### Early postoperative functional performance analysis

The early postoperative period during the in-patient stay showed no significant differences in its duration (Table 3). The early postoperative X-ray controls taken commonly two days after surgery showed that leg length in the AMIS group was equal within the range of ± 5 mm compared to the contralateral side significantly more often than in the LCS group (Table 3). Although only 5 patients in the AMIS group were able to reach full weight-bearing during their in-patient stay, this was significantly more frequent than in the LCS group, in which no patient reached full weight-bearing in this early period after surgery. While most patients of both groups reached a level of mobility to a standing position during physiotherapy in a similar time frame, significantly more patients could walk with a rollator or less support in the AMIS group compared to patients of the LCS group (Table 3). Moreover, of those patients being able to walk with a rollator or less support, patients of the AMIS group reached this level of mobilization significantly earlier (Table 3). The type of walking aids was forearm crutches in most of the cases in the AMIS group, while in the LCS group most patients still required a rollator or walking frame (Table 3).

**Table 3:** Analysis of early postoperative functional outcome of patients suffering from femoral neck fracture (FNF) and treated by either anterior minimal-invasive surgery (AMIS) or lateral conventional surgery (LCS) for hip hemiarthroplasty

	AMIS (*n* = 50)	LCS (*n* = 50)	*p* value
Duration of in-patient stay [day]	13.3 ± 0.9	13.1 ± 0.6	0.43^a^
Radiological leg length discrepancy [mm]			0.03^b^
Equal	36*	25*	
Unequal	11*	20*	
Full weight-bearing during in-patient stay possible	5*	0	0.03^b^
Mobilization to standing position during in-patient stay [postoperative day]	3.0 ± 0.5	3.1 ± 0.3	0.13^a^
Mobilization with rollator or less support during in-patient stay			
Possible	23*	13*	0.02^b^
If possible, on which postoperative day	5.1 ± 0.5*	6.9 ± 0.6	0.03^a^
Need for walking aids at discharge			0.11^b^
None	0	0	
Forearm crutches	16	10	
Rollator / walking frame	22	34	
Wheelchair	5	3	
Bedridden	7	3	

Mean ± SEM; *p < 0.05

^a^Mann–Whitney *U* Test

^b^Chi-square test/Fisher exact test

### Functional performance and surgery-related analysis 12 months after surgery

At 12 months after surgery, clinical investigation of the functional performance showed that most patients in both groups had improved their level of mobility (Table 4). No significant differences could be observed between the two groups (Table 4). Despite few bedridden patients, 18 patients (AMIS) and 17 patients (LCS) were able to stand and of those, most of them achieved full weight-bearing during walking (AMIS: 17; LCS: 17; *n* = 20 each group) and were able to take stairs (AMIS: 16; LCS: 16; *n* = 20). Radiological signs of sintering were observed in only 1 patient and was most likely due to choosing a too small implant. Analysis of the complication rate showed no significant differences between the two groups (AMIS: 14; LCS: 16; *n* = 26 in each group). The occurred complications were mainly independent of the surgery and rather due to the reduced medical health status of the geriatric patients in general (Table 4).

**Table 4:** Analysis of functional performance outcome 12 months postoperatively and analysis of complication rate within the first 12 months after surgery of patients suffering from femoral neck fracture (FNF) and treated by either anterior minimal-invasive surgery (AMIS) or lateral conventional surgery (LCS) for hip hemiarthroplasty

	AMIS	LCS	*p* value
Radiological sintering [mm]	*n* = 20	*n* = 20	0.31^a^
None	19	20	
Positive	1	0	
Full weight-bearing possible	17	17	1.0^a^
Mobilization to standing position possible [number of patients]	18	17	0.63^a^
Walking on stairs possible [number of patients]	16	16	1.0^a^
Need for walking aids [number of patients]			
None	8	7	0.74^a^
Improved compared to early in-patient analysis	12	10	0.52^a^
Complications within 12 months after surgery	n = 26	n = 26	0.84^a^
None	14	16	
Surgery-related	2	2	
Others not related to the surgery	10	8	

^a^Chi-square test / Fisher exact test

## Discussion

The present study demonstrates that AMIS for bipolar hemiarthroplasty of the hip after displaced fracture of the femoral neck is more beneficial perioperatively and in the early postoperative phase compared to the conventional lateral surgical approach in geriatric patients. This is indicated by reduced blood loss as well as reduced soft tissue injury intraoperatively without the increased duration of the procedure, by more patients achieving full weight-bearing of the injured leg after AMIS compared to LCS and by more patients being able to walk with a rollator or less support during their in-patient stay. Moreover, of those being able to walk with walking aids, patients of the AMIS group achieved mobility earlier than those of the LCS group, although their previous walking distance before the acute injury was comparatively reduced already.

Hemiarthroplasty or total arthroplasty of the hip are the most common procedures in geriatric patients suffering from a displaced FNF. However, the choice of arthroplasty remains controversial [8, 9]. In general, THA is recommended for rather active elderly, whereas BHHA is usually performed in geriatric patients with reduced health status [10, 11]. Registry data further support that for both procedures cemented fixation in geriatric patients is recommended [11]. Accordingly, we herein performed BHHA in patients with a reduced health status, which is characterized by an ASA score ranging mainly between II and III and by the fact that the majority of the patients had a limited walking distance in both groups before the injury.

Despite the choice of arthroplasty after FNF, several surgical approaches to the hip can be performed for arthroplasty. To date, the minimal-invasive anterior approach shows increasing popularity, whereas the conventional lateral and posterior approach have repeatedly shown solid outcomes [12, 13]. No surgical approach has shown clear superiority over the others so far and recommendations are not always clear to follow due to the variety of different results. Therefore, it has been postulated that the choice of approach should be based on the surgeon’s experience, the surgeon’s and patient’s preferences and patient characteristics [14]. However, in geriatric patients with reduced health status, it seems reasonable that the decision is not made upon the surgeon’s experience and personal preferences, but instead put the patient in the center of the decision and make every effort to reduce the grade of invasiveness of the procedure for these vulnerable patients. For this purpose, surgery-related factors such as the surgical approach reducing the perioperative strain on the patient should be considered. The present study demonstrates that in the AMIS group intraoperative blood loss was significantly reduced and the complication rate was similar compared to the well-established lateral approach. Also, the surgical intervention in the AMIS group did not last longer than in the LCS group. Moreover, within the first 13 days after surgery (approximate mean in-patient stay in both groups) the level of mobility was better in patients receiving AMIS as indicated by the fact that more patients only needed a rollator or less support. Although more patients in the AMIS group achieved full weight-bearing in the early postoperative phase, this amount of patients was low in both groups. This may be due to the early observation time point after surgery and a decreased resilience in the study population of geriatric patients with a reduced health status independent from the acute trauma. Further, patients of the AMIS group achieved this functional level sooner after surgery (approximately five (AMIS) versus seven (LCS) days postoperatively). Of interest, the improved outcome in patients after AMIS could be achieved, although the walking distance in this group was lower than in patients of the LCS group and only 19/50 patients (AMIS) compared to 28/50 patients (LCS) had no need for walking aids before the acute injury. Thus, functional recovery after the minimal-invasive approach appears to be faster and better in the early postoperative phase compared to the lateral approach to the hip in the present study, whereas 12 months after surgery no significant differences in the functional and surgery-related outcome can be observed. This may be due to the fact that the surgeon follows anatomical lines between the different muscle layers during the anterior approach instead of dissecting functionally important muscle tissue as in the lateral approach. These results are in line with a previous study analyzing primary total hip arthroplasty via the anterior approach versus the lateral approach and with other most recent studies analyzing the anterior approach to the hip for arthroplasty in trauma surgery [15–17]. Therefore, we believe that geriatric patients with displaced FNF should primarily be treated by AMIS to reduce the perioperative strain on the patient as well as accelerate the reconvalescence postoperatively. In this context, it should be considered that FNF is a common injury with rising prevalence worldwide that is currently being treated ubiquitously in many hospitals [18]. It may be assumed that BHHA via AMIS is beneficial for patients if performed exclusively in hospitals, that are focusing on this procedure and can ensure that surgery is performed within the first 24 h after injury to keep the complication rate as low as possible [19].

The focus of this study is on the effect of different approaches on the perioperative and early postoperative outcome after BHHA. Although we also provide data at 12 months after surgery demonstrating that AMIS does not appear to be functionally superior compared to LCS at this late time point, the long-term analyses remains a limitation of this study. However, including more patients or even longer time points postoperatively for follow-up, may be difficult in this geriatric, often multimorbid population. In addition, the use of different implants for each surgical approach may have affected the functional outcome of the two groups. However, no implant failure was observed in any group and surgery-related complications were independent of the choice of implant. Therefore, we feel that this limitation did not affect the conclusion of the study.

## Conclusion

In conclusion, geriatric patients with displaced fractures of the femoral neck treated by bipolar hemiarthroplasty via AMIS undergo less surgery-related strain and recover faster in the early postoperative phase than patients receiving BHHA via a lateral surgical approach. The choice of surgical approach should be made upon the patient’s characteristics and not on other parameters as e.g. the surgeon’s ability. Therefore, primary treatment via AMIS is recommended in these vulnerable patients and should be performed in specialized hospitals without any avoidable delay.
